# Comparative Effectiveness of After-School Programs to Increase Physical Activity

**DOI:** 10.1155/2013/576821

**Published:** 2013-08-04

**Authors:** Sabina B. Gesell, Evan C. Sommer, E. Warren Lambert, Ana Regina Vides de Andrade, Lauren Whitaker, Lauren Davis, Bettina M. Beech, Stephanie J. Mitchell, Nkiruka Arinze, Stevon Neloms, Colleen K. Ryan, Shari L. Barkin

**Affiliations:** ^1^Department of Social Sciences and Health Policy, and the Maya Angleou Center for Health Equity, Wake Forest School of Medicine, Winston-Salem, NC 27157, USA; ^2^Department of Psychology and Human Development, Vanderbilt University, Nashville, TN 37240, USA; ^3^Kennedy Center, Vanderbilt University, Nashville, TN 37203, USA; ^4^Department of Economics, Center for Evaluation and Program Improvement, Peabody College, Vanderbilt University, Nashville, TN 37203, USA; ^5^Clinical Research Center, Vanderbilt University, Nashville, TN 37232, USA; ^6^Center for Evaluation and Program Improvement, Peabody College, Vanderbilt University, Nashville, TN 37203, USA; ^7^Departments of Family Medicine and Pediatrics, Office of Rural Health and Health Disparities, University of Mississippi Medical Center, Jackson, MS 39216, USA; ^8^Center for Translational Science, Children's National Medical Center, Washington, DC 20010, USA; ^9^Vanderbilt University School of Medicine, Nashville, TN 37232, USA; ^10^Nashville Metropolitan Board of Parks and Recreation, Nashville, TN 37215, USA; ^11^Department of Pediatrics, Vanderbilt University School of Medicine, Nashville, TN 37232, USA

## Abstract

*Background*. We conducted a comparative effectiveness analysis to evaluate the difference in the amount of physical activity children engaged in when enrolled in a physical activity-enhanced after-school program based in a community recreation center versus a standard school-based after-school program. *Methods*. The study was a natural experiment with 54 elementary school children attending the community ASP and 37 attending the school-based ASP. Accelerometry was used to measure physical activity. Data were collected at baseline, 6 weeks, and 12 weeks, with 91% retention. *Results*. At baseline, 43% of the multiethnic sample was overweight/obese, and the mean age was 7.9 years (SD = 1.7). Linear latent growth models suggested that the average difference between the two groups of children at Week 12 was 14.7 percentage points in moderate-vigorous physical activity (*P* < .001). Cost analysis suggested that children attending traditional school-based ASPs—at an average cost of $17.67 per day—would need an additional daily investment of $1.59 per child for 12 weeks to increase their moderate-vigorous physical activity by a model-implied 14.7 percentage points. *Conclusions*. A low-cost, alternative after-school program featuring adult-led physical activities in a community recreation center was associated with increased physical activity compared to standard-of-care school-based after-school program.

## 1. Introduction 

Childhood obesity remains one of the most serious threats to the public's health, with 1 in 3 children and adolescents overweight or obese (body mass index (BMI) ≥ 85th percentile) [1]. Childhood obesity is particularly problematic because it is resistant to treatment once established [2]. Accordingly, public health authorities are focusing on prevention. There is limited evidence for effective behavioral prevention interventions [3]. To fill this gap, the Institute of Medicine [4], the *Strategic Plan for NIH Obesity Research* [5], Shaping America's Youth [6], and the White House Task Force on Childhood Obesity [7] have called for community-engaged, family-centered approaches to pediatric obesity prevention. These approaches are thought to have the greatest potential for sustained efforts and effects in our obesogenic environment. 

In parallel, comparative effectiveness research is being discussed within the national health reform debate as a mechanism for improving healthcare quality and decreasing healthcare spending [8]. Clinical research typically examines the effectiveness of one prevention or treatment method at a time. Comparative effectiveness research compares multiple methods to determine the effectiveness of an intervention relative to alternatives. Identifying the most effective and efficient interventions has the potential to reduce unnecessary treatments, which should lower costs. 

It is estimated that 8.4 million children attend after-school programs (ASP), and an additional 18.5 million would do so if a program was available [9]. The purpose of this study was to evaluate the comparative effectiveness of a community-driven ASP designed to combat physical inactivity versus a standard-of-care school-based ASP available to working parents. The community ASP was derived directly from local input and sustained through the collaboration and sharing of resources by the parks department and the public school system (Davidson County, TN, USA). This community-engaged effort has the potential to serve as a new model for youth obesity prevention because (1) it systematically addresses the top four barriers, identified by Shaping America's Youth, that prevent children from being active (lack of access to safe and appropriate places to be active, parental time constraints, cost of programs, and lack of parental motivation [6]) and (2) it engages multiple sectors of society to support program attendance and sustainability. 

There is limited published research on ASPs designed to increase physical activity. Systematic reviews suggest that it is possible to improve activity levels, physical fitness, body composition, and blood lipids in the after-school setting [10] and that limitations in study design, lack of statistical power, and problems with implementation have hindered the evaluation of most ASPs to date [11]. 

To assess the comparative effectiveness of this community-driven ASP as a pediatric obesity prevention intervention, we compared it to the routine aftercare available to working parents in the community and asked two research questions: (1) Are children in the alternative ASP more physically active than children in the standard ASP? (2) Do the operating costs associated with these programs differ?

## 2. Materials and Methods

This study was guided by principles of community-based participatory research (CBPR). CBPR is an important research approach that equitably involves community members who are affected by the issue being studied in all phases of the research process [12]. The community ASP was developed by the parks department to address the community's need for an affordable ASP. Families provided input about their needs and preferences (e.g., transportation from school to the community center, flexibility in pick-up times, homework time, reduction of screen time, and increased physical activity). The city's public school system changed its policy around permissible bus stops allowing buses to deliver students to the community recreation center to support program attendance. The leadership and staff of the parks department were involved in all aspects of this research project: grant acquisition, study design, implementation, interpretation of results, and dissemination.

### 2.1. Study Population and Design

The study design was an observational prospective cohort study and a natural experiment in Nashville, TN, USA. The “naturally occurring” event, the parks department's new ASP, was the intervention, and children attending this community ASP formed the intervention group (*N* = 54). Comparison participants (*N* = 37) were recruited from an ASP located in the same (low income) school district and operated by a national company that operates a high proportion of school-based ASPs in the city, making it de facto standard-of-care for the majority of the city's school-aged children with working parents. Children were eligible for the study if the following was true: (1) age ≥5 and <13 years; (2) attended one of the Glencliff [neighborhood] cluster of public elementary or middle schools; (3) enrolled in the community or school-based ASP. Parents of eligible children underwent a 15-minute oral consent process before providing written consent for their child. Children provided assent. The consent/assent process was conducted in the preferred language of English or Spanish. The study was approved by the Vanderbilt University Institutional Review Board (#090986). 

The two ASPs followed similar formats, and operated from 3–6 PM every day public schools were open. Both ASPs included time for snack, homework, and play and did not focus on a single activity (e.g., tutoring, chess, and team sport). The community ASP was set in a community recreation center and involved staff-led games. The school-based ASP was set in a school cafeteria and involved opportunities for arts and crafts and playing on the playground. The main differences between the two ASPs were (1) format of active play time (adult-led versus unstructured) and (2) location (community recreation center versus public school). Refer to Table 1 for a direct comparison of ASP structure and process.

### 2.2. Data Collection

All measures were collected at the ASPs at three time points over approximately 12 weeks (February–May 2010), with six weeks separating each wave of measurement. The measurement period was selected based on the Cochrane Review that states that obesity prevention interventions should last at least 12 weeks for behavior change to be observed [13]. 

### 2.3. Measures

#### 2.3.1. Physical Activity

Physical activity was assessed using ActiGraph GT1M accelerometers (ActiGraph, Pensacola, FL, USA) only during ASP programming time. Accelerometry is considered an objective measure of physical activity [14] and has been used with children [15, 16], including Latino and African-American children, with high reliability: *r* = 0.93 [17]. The ActiGraph is a small monitor that is worn on an elastic waist belt and measures the intensity of physical activity associated with locomotion. Monitors were programmed to record in continuous 10-second epochs to capture the short, spurt-like activity characteristic of children. At each measurement period, the children wore monitors for five consecutive days, from the time they signed into the ASP until they were picked up. Measurement start and stop times were recorded by study staff at each site; these were used as precise wearing cut-off points, eliminating the need for wearing/nonwearing time analysis. Data were retained in analysis if the child wore the accelerometer a minimum of 3 days of the given measurement period [18, 19]. 

Freedson's age-dependent cut points were used to determine time spent in sedentary, light, moderate, and vigorous activity [20]. Trost's validation study comparing various accelerometer cut points for predicting physical activity in children supports the application of Freedson equations in field-based studies of school-aged children. In particular, Trost found that, for classification of MVPA (moderate-vigorous physical activity), Freedson cut points exhibited excellent classification accuracy [21]. The analyses described below were also conducted using Pate's cut points and resulted in similar findings (not reported here) [22]. 

Daily percentage of time spent in each level of physical activity (i.e., sedentary, light, moderate, and vigorous) was determined by dividing the minutes spent in each activity level by the sum of minutes the ActiGraph was worn in a day (i.e., time in attendance at the ASP). Children spent varying amounts of time in ASPs depending on their family needs. Thus, the continuous outcome measures were the proportion of time spent in LMVPA (light-moderate-vigorous physical activity) or MVPA (moderate-vigorous physical activity) out of total time in attendance, rather than the number of minutes the program was open, to allow for a meaningful comparison within individuals and across groups. Daily percentages were averaged across days to create individual participants' physical activity (PA) scores at each measurement period.

#### 2.3.2. Body Mass Index (BMI)

Body weight was measured after voiding while children wore light clothing without shoes. Calibrated digital scales (Detecto, Webb City, MO, USA, Model#758C) were accurate to the nearest 0.1 kg. Body height without shoes was measured to the nearest 0.1 cm with the scale's stadiometer. BMI percentile, adjusted for age and gender, was calculated using these measurements [23]. Weight categories were defined by BMI percentile, according to Centers for Disease Control growth charts: underweight: <5th percentile; healthy weight: 5th to <85th percentile; overweight: 85th to <95th percentile; obese: ≥95th percentile [23]. 

#### 2.3.3. Body Fat Percentage

Body composition was measured by the RJL Systems BIA Quantum II (RJL Systems, Clinton, MI, USA) after voiding. Standard procedures for whole body bioelectrical impedance measurement were used [24], along with the vendor-provided child-specific regression equation to estimate percent fat mass from total body water. 

#### 2.3.4. Fitness

Children were asked to complete a 1/2 mile run as fast as possible on a running track [25]. Time of completion was recorded to the nearest second. 

#### 2.3.5. Demographics

Parents completed a survey asking about child's date of birth (used to calculate age), gender, race/ethnicity, and name of school.

### 2.4. Statistical Analysis 

#### 2.4.1. Analysis of Preexisting Site Differences

Because children were not randomly assigned, preexisting differences between groups were potential confounders. Therefore, we compared children enrolled in the ASPs to test for differences on basic demographic and process variables, using bootstrap *t*-tests that controlled for the familywise false discovery rate [26]. 

#### 2.4.2. Physical Activity Data Analysis

To assess change in PA over time, a conditional linear latent growth model was used with random intercepts and slopes that were free to covary and time varying error variances. The model was estimated using Mplus version 6.11 [27]. This approach offers important advantages over older analysis of variance (ANOVA) models [28], such as (a) better accuracy in assessing change over time, (b) graceful handling of missing values and unequal time intervals between waves and participants, and (c) repeated measurements that increase statistical power [29]. The key result is a group by time interaction, which shows whether groups differ in their slopes/rates of change in PA. Centering time zero at the first measurement let us answer two questions: (1) Did the groups start out equally? And (2) did their time slopes differ? The analysis assumed data were missing at random and used full information maximum likelihood to maximize sample size by including all participants with at least one wave of data.

#### 2.4.3. Cost-Effectiveness Analysis

We used the cost analysis guidelines for research evaluation proposed by Levin and McEwan [30]. Using the ingredient method, we estimated the implementation costs, without estimating indirect costs or externalities associated with the programs, to indicate how much it would cost to replicate each ASP. Instead of accounting expenditures paid during the implementation, we valued resources using standard costs to society. All personnel time (including volunteer time) was valued by using the median earning per hour of a comparable worker published by the Bureau of Labor Statistics 2010 [31]; thus, differences in human capital endowment did not affect our estimates of implementation costs.

## 3. Results and Discussion

Of the 91 children who attended the ASPs, baseline demographics were obtained from parents of 83 children. The analytic sample included the 82 children with PA data from at least one time point; one child in the school-based ASP did not provide at least 3 days of PA data in any measurement period and was not included in the analyses. Of the 82 participants, 62 had data for all three time points, 16 had data for two time points, and 4 had data for only one time point.

### 3.1. Demographics and Process Measures

The baseline sample was 65% female and 7.9 years of age (SD = 1.7) on average; 57% were healthy weight, 23% overweight, and 20% obese; 40% were African-American, 40% White, and 20% Latino. On average at baseline, children spent 77.4% (SD 10.3%) of the ASP in LMVPA and 27.5% (SD 14.3%) in MVPA. At baseline, children in the two ASPs did not differ in gender, age, BMI, percent body fat, fitness (Table 2), or physical activity level (Table 3). However, children in the community ASP were less likely to be white than children in the school-based ASP (*P* = .027, Table 2). At baseline, children spent approximately 30% of their ASP time in MVPA (SD = 15.6).

### 3.2. Change in Physical Activity over Time

**Figure 1:**
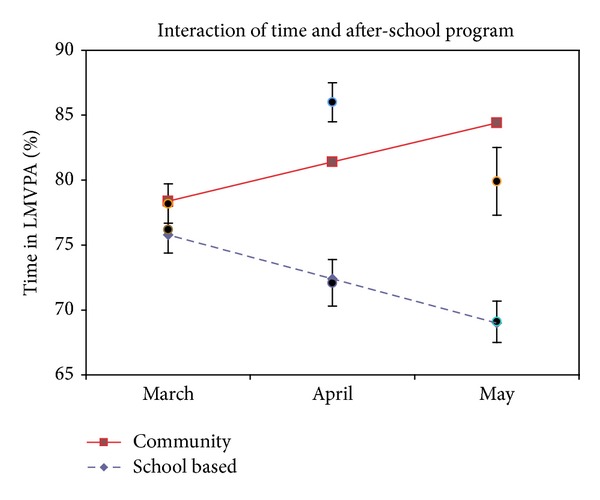
Percent of time spent in physical activity (LMVPA) after-school. Notes: lines show mixed model outcome slopes; points show observed means ± standard error.

**Figure 2:**
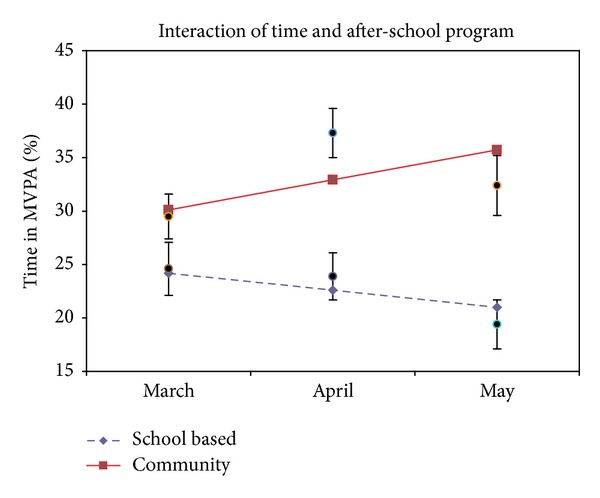
Percent of time spent in moderate/vigorous physical activity (MVPA) after-school. Notes: lines show mixed model outcome slopes; points show observed means ± standard error.

The linear latent growth model implied that, on average, children in the community ASP became more active over time (average change between Baseline-Week 6 and Week 6-Week 12), compared to the children in the school-based ASP (Table 3). Children in the school-based ASP reduced their total physical activity (LMVPA) by an average of 3.4 percentage points over each measurement period (*P* = .002), for a total 6.8 percentage point decrease over the 12-week study period. In contrast, children in the community ASP increased their total physical activity (LMVPA) by an average of 3.0 percentage points over each measurement period (*P* = .006), for a total 6 percentage point increase over the 12-week study period (Figure 1). Most of this increase in activity was in high intensity activity. Children in the school-based ASP did not significantly change their MVPA on average (*P* = .12). However, children in the community ASP increased their MVPA by an average of 2.8 percentage points over each measurement period (*P* = .006), for a total 5.6 percentage point increase over the 12-week study period (Figure 2). Taken together, the model-implied average difference between the two groups of children at Week 12 was 15.4 percentage points in LMVPA (*P* < .001) and 14.7 percentage points in MVPA (*P* < .001), favoring the activity-enhanced community ASP. However, as a more conservative indicator, the observed average difference between the two groups of children who had data at Week 12 was 10.8 (*P* = .001) percentage points in LMVPA and 13 percentage points in MVPA (*P* < .001). 

### 3.3. Cost-Effectiveness Analysis

The community ASP served 54 children; the school-based ASP served 37 children. Total implementation costs (valued in 2010 dollars) for the 12-week study period were $1,184 per child ($19.25 daily per child) for the community ASP, compared to $1,087 per child ($17.67 daily per child) for the school-based ASP (9% difference; Table 4). The facility cost represented 66% and 65% of the total implementation costs to run the community ASP and school-based ASP, respectively. The main source of cost differential between programs was the child to staff ratio (6 : 1 at the community ASP, 12 : 1 at the school-based ASP). To run the ASPs for 12 weeks, the personnel cost was $380 per child for the community ASP compared to $314 per child for the school-based ASP (21% difference). 

### 3.4. Implications

With more than 23 million parents of school-aged children employed full-time [32], ASPs are ideal for systematic interventions to increase physical activity. This study demonstrated that, compared to a standard-of-care school-based ASP, an ASP set in a community recreation center with activities directed by recreation staff significantly increased total physical activity in a multiethnic sample of public school children by 6 percentage points over 12 weeks. Most (5.6%) of this increase was in MVPA, which is the type of physical activity that has the greatest health benefits [33–35]. The incremental cost of implementing the activity-enhancing ASP compared to the traditional ASP was $1.59 per day per child. The main source of cost differential between programs was their child to staff ratios. 

Assuming the improvement in activity was solely due to the intervention; these findings suggest that children attending traditional school-based ASPs, already costing an average of $17.67 per day, would need an additional daily investment of $1.59 per child over 12 weeks to increase their LMVPA by 15.4 percentage points or their MVPA by a model-implied 14.7 percentage points. Cost-effectiveness analyses are often lacking for community-based prevention efforts. The annual cost of childhood obesity-related health expenses in the US is $14.1 billion for outpatient care and $237.6 million for inpatient care which translates to about $5 in healthcare expenses per day per child, without including other relevant long-term costs related to school performance, labor market involvement, quality of life, welfare needs, and so forth [36]. Given this, providing structured PA programming by qualified staff in a community recreation center in the after-school hours could be a reasonable low-cost investment. 

ASPs have long played a critical role in supporting academic achievement, safety, discipline, and avoidance of risky behaviors [37]. They could now be leveraged as part of a broader approach to address physical inactivity. Community centers operated by local parks and recreation departments (20,000 nationally) provide an ideal venue for structured PA programming for children [38] in large part because these centers, in conjunction with school transportation departments, can address community-based barriers [6] to increasing children's activity levels. It is noteworthy that the parks staff initiated and led this program on their own. Their intimate knowledge of the community and the respect they commanded from both the children and adults in the community likely contributed to the program's success. We speculate that the combination of the built environment that supported activity, low child to staff ratio, and intentional activity leadership resulted in the increased PA levels. The school-based ASP could have let children play in the school's gymnasium if there had been additional staff available to supervise. Thus, we speculate that importing adult-led activities with lower child to staff ratios into school-based ASPs might be a cost-effective approach to increasing activity in that setting as well. This would need to be tested.

### 3.5. Limitations

First, accelerometers do not adequately measure body movements of upper and lower extremities, but they are considered the gold standard for measuring PA under free-living conditions. This should not have biased our results since the limitation of accelerometry was the same across groups. Second, our sample was small but having three waves of data increased statistical power and was sufficient for detecting a significant increase in PA under free-living conditions. Third, despite efforts to select a comparable comparison group and measure potential confounders, we cannot rule out all systematic differences between the two groups. We did rule out the most important possible confounds in the literature: body composition, fitness, age, and gender. It is possible that the difference in racial composition of the groups could explain baseline variance [39] but is unlikely to explain change over time in activity levels. 

Fourth, for the community ASP, there were significant differences between the observed and model-implied averages at Weeks 6 and 12. These discrepancies highlight the fact that the final specified model did not perfectly recreate the observed data. This could have been partially due to missing data for this group at either time point. The discrepancies could also have arisen because the community ASP's growth rate was not linear; yet, a model with only three time points does not have the degrees of freedom to investigate more sophisticated growth parameter specifications (e.g., quadratic). Nonetheless, applying latent growth models has provided further insight into how ASPs might impact children's PA over time (e.g., what effect does ASP type have on PA change over time? What is the typical growth rate of PA for children over time? Do some programs increase the growth rate of certain types of PA (e.g., light, moderate, or vigorous) more than others? What is the functional form of PA change over time?) 

## 4. Conclusion

An ASP set in a community recreation center and led by recreation staff incorporating structured physical activity opportunities was associated with significant increases to physical activity during ASP time in a multiethnic sample of public school children in 12 weeks, compared to a standard school-based ASP. Utilizing community recreation centers' built environment and staff could be a promising low-cost proposition to improve health trajectories among school-aged children. 

## Figures and Tables

**Table 1:** Comparison of after-school programs.

	Community (intervention)	School-based (comparison)
Location	(i) Community recreation center	(i) Public school cafeteria

Who	(i) Ages 5–14 yrs	(i) Only open to students at that elementary school (5–10 yrs)

Program format	(i) 3–6 PM	(i) 3–6 PM
	(ii) Transportation from neighborhood public schools to the community center (iii) Parents pickup from the community center	(ii) Transportation not necessary (iii) Parents pickup from school
	(iv) Snacks provided	(iv) Snacks provided
	(v) Homework help provided	(v) Homework help provided
	(vi) Staff-led activities (children select activity)	(vi) Unstructured play time (children select activity)

Stated physical activity goal	(i) 60 minutes of activity/day	(i) 45 min of moderate activity 3/week (ii) 45 min of vigorous activity 2/week

Physical activities (always available)	(i) Staff leads students through activities: (a) basketball scrimmage, (b) dance, (c) cross country, (d) swimming, (e) recreational games (e.g., flag tag, 4 square, and scooter relays)	(i) Staff supervises for safety: (a) playground, (b) gymnasium

Nonphysical activities (always available)	(i) Arts and crafts	(i) Arts and crafts (ii) Reading (iii) Board games, blocks

Physical activity resources (used during the after-school program)	(i) Playground (ii) Gymnasium with basketball court (iii) 2 playing fields (iv) Running trail (v) Swimming pool	(i) Playground

Cost	(i) Free of cost to families (ii) Department of Parks and Recreation assumed operational costs (iii) Public school system assumed transportation costs	(i) $46.50/week paid by family (ii) Financial assistance available

**Table 2:** Between-group comparison of baseline and process measures.

	Community ASP (*n* = 47)				School-based ASP (*n* = 36)				P*	
	Mean/%	SD	Min	Max	Mean/%	SD	Min	Max	*P*_raw_	*P*_boot_
Child characteristics										
Male	43%				25%				0.10	0.44
Hispanic ethnicity	26%				11%				0.10	0.45
Black	47%				31%				0.14	0.57
White	26%				56%				**0.005**	**0.027**
Age at baseline (yrs)	8.79	1.67	5.57	12.08	7.96	1.55	5.45	10.34	0.023	0.12
BMI percentile**	74.74	23.60	8.40	99.60	73.87	21.03	11.60	99.40	0.86	1.00
Body fat percentage	29.26	11.27	5.80	54.30	29.92	8.08	15.90	48.60	0.77	1.00
Fitness (1/2 mile run time in min)	6.29	1.09	4.23	9.41	6.08	1.13	4.23	8.51	0.40	0.99

Process measures										
Waves of data collection per child	2.83	0.52	1.00	3.00	2.92	0.37	1.00	3.00	0.40	0.94
Minutes activity monitor worn per measurement period	108.74	20.37	57.60	143.00	105.83	27.02	33.20	149.25	0.59	1.00

^∗^To control for multiple testing we show the raw probability of alpha along with a bootstrap simultaneous alpha based on 100,000 resamples with replacement.

**Underweight: <5th percentile; healthy weight: 5th to <85th percentile; overweight: 85th to <95th percentile; obese: ≥95th percentile.

**Table 3:** Between-group comparison of time spent in physical activity (model-implied estimates).

	Community ASP		School-based ASP		Group difference	
	% Time at activity level	*P* (difference from 0)	% Time at activity level	*P* (difference from 0)	% Time at activity level	*P* (for group difference)
Baseline						
LMVPA	78.4	<0.001	75.8	<0.001	2.6	0.30
MVPA	30.1	<0.001	24.2	<0.001	5.9	0.06

Change per measurement period (6 weeks)*						
LMVPA	3.0	0.006	−3.4	0.002	6.4	<0.001
MVPA	2.8	0.006	−1.6	0.12	4.4	0.002

*Average change between Baseline-Week 6 and Week 6-Week 12.

**Table 4:** Total implementation cost per participant and program (2010 dollars).

	Community ASP (serving 54 children with 9 staff members/volunteers)	School-based ASP (serving 37 children with 3 staff members)
Facilities** (space used in sq ft)	$781	$706

*Personnel* (hours per day, days worked, and hourly rate of staff/art teacher/volunteers)	$380	$314

Snacks	$17	$62

Recreational equipment*** (e.g., games, toys, sports gear, and art supplies)	$6	$4

Total direct cost per participant for 12 weeks	$1184	$1087
Daily direct cost per participant	$19.25	$17.67

**The facilities used by the community ASP covered a larger area compared to those used by the school-based ASP (111,130 sq ft, versus 68,940 sq ft). The space available for these programs was valued based on $1 per sq ft, assuming participants used 50% of the available space while the other 50% continued to be available to the public. We estimated the cost of using the facilities during 3 hours per day over the 61.5 days of study period.

***The school-based ASP reported a 100% depreciation rate within one year. The community center program also reported 100% depreciation rate for light recreational equipment within one year and a 5-year life span on electronics and large equipment.
